# Interleukin-13 R130Q aggravates airway smooth-muscle phenotypic switching and promotes airway remodeling in severe asthma

**DOI:** 10.3389/fphys.2026.1906184

**Published:** 2026-08-25

**Authors:** Yafang He, Jingyi Sun, Luanluan Li, Shasha Bai, Xiaojia Zhai, Ye Tian, Qin Pan, Yixiao Bao, Guodong Ding

**Affiliations:** 1 Department of Pediatric Pulmonology, Xinhua Hospital affiliated to Shanghai Jiao Tong University School of Medicine, Shanghai, China; 2 Department of Developmental and Behavioral Pediatrics, Shanghai Children’s Medical Center, Shanghai Jiao Tong University School of Medicine, Shanghai, China; 3 Shanghai Institute of Pediatric Research, Shanghai Key Laboratory of Pediatric Gastroenterology and Nutrition, Xin Hua Hospital, Shanghai Jiao Tong University, School of Medicine, Shanghai, China; 4 School of Health Science and Engineering, University of Shanghai for Science and Technology, Shanghai, China; 5 Department of Integrative Medicine for Allergy, Shanghai Tonxin Pediatric Clinic, Shanghai, China

**Keywords:** airway remodeling, airway smooth-muscle cell, asthma, IL-13 R130Q, phenotypic switching

## Abstract

**Introduction:**

Airway remodeling represents a key pathological feature of severe asthma. Although the interleukin-13 (IL-13) R130Q variant is associated with increased disease severity, its specific contribution to airway remodeling remains unclear.

**Methods:**

This study used an ovalbumin (OVA)-induced asthma model in which mice were sensitized intraperitoneally with OVA/alum and subsequently challenged intranasally with OVA; human IL-13 130R or IL-13 130Q was administered intranasally before each challenge. Primary human bronchial smooth muscle cells (hBSMCs) from a single healthy donor were treated with both variants to assess CCK-8-derived proliferative/metabolic activity, migration, acetylcholine (ACh)-induced intracellular calcium responses, and IL-4 and IL-5 concentrations in culture supernatants. JAK2 and STAT6 phosphorylation was evaluated separately as a downstream signaling readout. Structural modeling and independent triplicate 100-ns molecular dynamics simulations of the IL-13/IL-13 receptor alpha 2 (IL-13Rα2) complex were performed using GROMACS 2025.4 with the AMBER ff19SB force field, and molecular mechanics/generalized Born surface area analysis was used for relative comparison of binding free energies.

**Results:**

IL-13 130Q produced limited airway changes in mice without OVA sensitization, but exacerbated airway wall thickening, luminal narrowing, smooth-muscle expansion, and pulmonary inflammation in OVA-sensitized and intranasally challenged mice. In hBSMCs, IL-13 130Q induced greater CCK-8-derived proliferative/metabolic activity, migration, ACh-induced intracellular calcium responses, and IL-4 and IL-5 concentrations than IL-13 130R at 10 -100 ng/mL. Molecular dynamics analyses suggested the reduced binding stability of IL-13 130Q/IL-13Rα2 complex, as reflected by fewer interfacial hydrogen bonds, altered intermolecular contacts, and a less favorable relative binding free energy. Elevated JAK2/STAT6 phosphorylation indicated the amplification of downstream signaling, mechanistically tied to potential reduced decoy-receptor affinity.

**Conclusion:**

These preclinical data support a contextdependent potentiating function of IL13 130Q in airway smoothmuscle phenotypic modulation and airway remodeling. Further investigation may rationalize genotype-based stratification in the therapy of severe asthma.

## Introduction

Asthma is a common chronic airway disease with an increasing incidence among children in recent decades. Although most patients respond well to corticosteroids and bronchodilators, approximately 5%–10% of adolescents respond poorly to conventional therapy, resulting in severe disease characterized by frequent wheezing, shortness of breath and/or chest tightness, progressive airflow limitation, and reduced lung function (Martin Alonso et al., 2017; Haktanir Abul and Phipatanakul, 2019; Manti et al., 2024). Biologic therapies targeting interleukin (IL)-13/IL-4 and IL-5 are often required to manage steroid-resistant severe asthma (Varricchi et al., 2022; Sahnoon et al., 2025). Therefore, early identification of patients who are likely to respond poorly to standard treatment may have important clinical implications.

Airway remodeling, characterized by structural alterations including epithelial injury, goblet cell hyperplasia or metaplasia, subepithelial extracellular matrix deposition and fibrosis, inflammatory cell infiltration and activation, and airway smooth muscle (ASM) cell hyperplasia or hypertrophy, is a major pathological hallmark of severe asthma (Liu et al., 2021; Joseph and Tatler, 2022; Hsieh et al., 2023; Sahnoon et al., 2025). Among the histopathological changes associated with airway remodeling, phenotypic switching of ASM cells plays a central role in disease initiation and progression. This process is characterized by increased proliferation, migration toward the epithelium, and enhanced secretion of pro-inflammatory mediators (Wright et al., 2013; Kardas et al., 2020). Consequently, ASM phenotypic switching represents a key pathological feature distinguishing severe asthma from mild-to-moderate disease (Pepe et al., 2005).

Accumulating evidence highlights the central role of IL-13, a T-helper 2 cytokine, in promoting ASM phenotypic switching. Previous studies have shown that IL-13 levels are elevated in patients with corticosteroid-resistant severe asthma compared with those with corticosteroid-sensitive asthma (Ntontsi et al., 2018). Functionally, IL-13 promotes pro-asthmatic responses in ASM cells through phosphorylation of signal transducer and activator of transcription 6 (STAT6) (Chiba et al., 2012). In addition, IL-13 enhances pro-asthmatic ASM phenotypes, including increased eotaxin release, augmented ASM contraction through upregulation of smooth muscle contractile proteins (Desai et al., 2011), activation of calcium signaling (Gao et al., 2010), and promotion of ASM cell migration (Parameswaran et al., 2007). Conversely, anti-IL-13 therapy has been shown to reduce asthma symptom severity and exacerbation frequency while improving lung function (Bagnasco et al., 2016).

The IL-13 R130Q variant is a relatively common single-nucleotide polymorphism in the IL-13 gene that results in substitution of arginine with glutamine at position 130. Our previous studies demonstrated that the R130Q variant exhibits significantly greater functional activity than wild-type IL-13 (Chu et al., 2012; He et al., 2013). Patients with asthma who are homozygous for the 130Q variant have significantly lower forced expiratory volume in one second, higher levels of inflammatory mediators (IL-13, IL-23, IL-11, and chemokine (C-C motif) ligand 8) in bronchoalveolar lavage fluid (BALF), and greater subepithelial thickness than those carrying the wild-type IL-13 allele (Nagashima et al., 2011). These findings suggest that the IL-13 R130Q variant may contribute to severe asthma by exacerbating airway remodeling through ASM phenotypic switching. However, evidence supporting this mechanism remains limited. Therefore, the present study aimed to investigate whether the IL-13 R130Q variant alters the ASM phenotype in a manner that promotes more severe asthma.

## Materials and methods

### Chemicals and reagents

Ovalbumin (OVA; grade V) and acetylcholine (ACh) were purchased from Sigma (Taufkirchen, Germany). Human enzyme-linked immunosorbent assay (ELISA) kits for IL-4 and IL-5 were obtained from RayBiotech (Peachtree Corners, GA, USA), whereas mouse ELISA kits for IL-4 and IL-5 were purchased from Beyotime (Jiangsu, China). Recombinant human IL-13 130R (wild-type IL-13) and IL-13 130Q were purchased from PeproTech (London, UK). Cell Counting Kit-8 (CCK-8) and Fluo-3/AM were obtained from Beyotime (Jiangsu, China). An RNA extraction kit and a complementary DNA (cDNA) synthesis kit were purchased from Takara Bio (Kusatsu, Japan), and quantitative polymerase chain reaction (qPCR) Master Mix was obtained from Yeasen (Shanghai, China). Primary hBSMCs (HBSMC, Cat. no. 3400) were commercially obtained from ScienCell Research Laboratories (Carlsbad, CA, USA), isolated from a single healthy human donor, and cryopreserved at passage 1. Cell-culture reagents were purchased from ScienCell.

### Animals

Female BALB/c mice aged 6–8 weeks were obtained from Shanghai Slack Laboratory Animal Co. (Shanghai, China) and used to establish an OVA-induced asthma model. The animals were housed under specific pathogen-free conditions at the Experimental Animal Center of Xinhua Hospital (24 ± 1 °C, 40%–70% relative humidity, 12-hour light/dark cycle) with free access to food and water. After acclimation, 48 mice were randomly assigned to six groups: control, IL-13 130R, IL-13 130Q, OVA, OVA pretreated with IL-13 130R (OVA + IL-13 130R), and OVA pretreated with IL-13 130Q (OVA + IL-13 130Q), with eight mice per group. All experimental procedures were approved by the Institutional Animal Care and Use Committee of Xinhua Hospital affiliated with Shanghai Jiao Tong University School of Medicine and were conducted in accordance with relevant institutional guidelines and regulations.

### Asthma induction and IL-13 treatment

**Figure 1:**
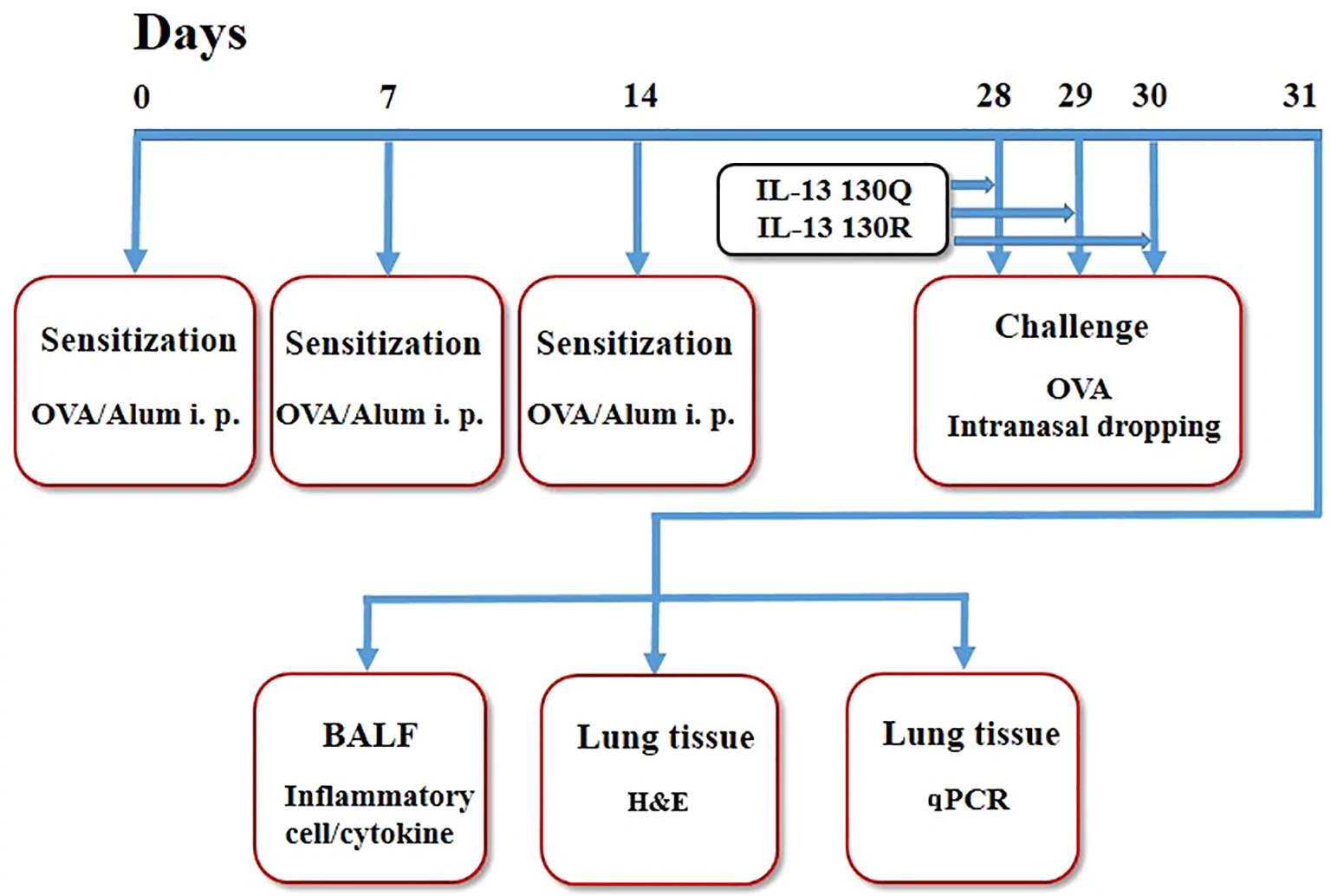
Schematic overview of the experimental design, showing induction of the OVA-induced asthma model and administration of IL-13 variants (IL-13 130R and IL-13 130Q) during the study. OVA, ovalbumin; IL-13, interleukin-13; BALF, bronchoalveolar lavage fluid; H&E, hematoxylin and eosin; qPCR, quantitative polymerase chain reaction.

The OVA-induced asthma model was established in two sequential phases (**Figure 1**) according to a previous report (Cho et al., 2006). During the allergic sensitization phase (days 0–14), mice in the OVA, OVA + IL-13 130R, and OVA + IL-13 130Q groups received intraperitoneal injections of 20 μg OVA in 0.1 mL aluminum adjuvant. Mice in the control, IL-13 130R, and IL-13 130Q groups received intraperitoneal injections of an equal volume of sterile phosphate-buffered saline (PBS). During the challenge phase (days 28–30), OVA-sensitized mice were challenged daily by intranasal administration of 100 μg OVA. Mice in the remaining groups received intranasal sterile PBS. Thirty minutes before each challenge, mice in the IL-13 130R, IL-13 130Q, OVA + IL-13 130R, and OVA + IL-13 130Q groups received intranasal administration of PBS containing 2.5 μg IL-13 130R or 2.5 μg IL-13 130Q, respectively, for three consecutive days. Mice in the control and OVA groups received an equivalent volume of PBS before each OVA challenge. The dose and route of administration for IL-13 130R and IL-13 130Q were based on previous reports (Chen et al., 2004; Chiba et al., 2012).

### Collection and counting of total BALF cells

Twenty-four hours after the final challenge, mice were anesthetized with isoflurane. A midline cervical incision was made to expose the trachea, followed by endotracheal intubation. Bronchoalveolar lavage fluid (BALF) was collected by lavaging both lungs twice through a tracheal cannula with 0.8 mL PBS, achieving a recovery rate of at least 80%. The lavage fluid was centrifuged at 1,500 rpm for 10 min at 2–8 °C, and the resulting cell pellet was resuspended in PBS for total BALF cell counting. Differential leukocyte counting and flow-cytometric phenotyping were not performed.

### Quantitative reverse transcriptase PCR (RT-qPCR)

Relative messenger RNA (mRNA) expression levels of cytokines (IL-13, IL-4, and IL-5) in lung tissue were measured by quantitative reverse transcription PCR (RT-qPCR) as previously described. In brief, 24 h after the final challenge, mice were anesthetized with isoflurane. A midline cervical incision was made to expose the trachea, followed by endotracheal intubation. Total RNA was isolated from one lung using the Takara MiniBEST Universal RNA Extraction Kit (Takara, Kusatsu, Japan) according to the manufacturer’s instructions. cDNA was synthesized from 500 ng of total RNA using PrimeScript RT Master Mix (Takara, Kusatsu, Japan). Gene expression was then analyzed using Hieff qPCR SYBR Green qPCR Master Mix (Yeasen, Shanghai, China) on an Applied Biosystems™ 7500 Real-Time PCR System. Relative gene expression was quantified using the 2^−ΔΔCt^ method with glyceraldehyde-3-phosphate dehydrogenase (GAPDH) as the reference gene. Melting curve analysis was performed to confirm the specificity of the PCR products. The primer sequences were as follows: IL-13 (forward: 5′-TGAGCAACATCACACAAGACC-3′; reverse: 5′-GGCCTTGCGGTTACAGAGG-3′), IL-4 (forward: 5′-GGTCTCAACCCCCAGCTAGT-3′; reverse: 5′-GCCGATGATCTCTCTCAAGTGAT-3′), IL-5 (forward: 5′-CTCTGTTGACAAGCAATGAGACG-3′; reverse: 5′-TCTTCAGTATGTCTAGCCCCTG-3′), and GAPDH (forward: 5′-GTCAAGGCCGAGAATGGGAA-3′; reverse: 5′-CTCGTGGTTCACACCCATCA-3′).

### IL-13 treatment of hBSMCs

Primary hBSMCs (HBSMC, Cat. no. 3400; ScienCell Research Laboratories, Carlsbad, CA, USA), isolated from a single healthy human donor and cryopreserved at passage 1, were cultured in smooth muscle cell medium (SMCM) supplemented with 2% fetal bovine serum, 0.5 ng/mL human epidermal growth factor, 5 μg/mL insulin, 2 μg/mL basic human fibroblast growth factor, 50 μg/mL gentamicin, and 50 ng/mL amphotericin B. Before treatment, cells were cultured in serum-free SMCM for 24 h and then assigned to IL-13 130R and IL-13 130Q groups, each subdivided according to IL-13 concentrations of 1, 10, 50, and 100 ng/mL (n = 4 per subgroup). Cells were incubated with the respective IL-13 variant for an additional 24 h. The concentration range and incubation period were based on a previous report (Syed et al., 2005).

### Cell proliferation assay

hBSMCs were seeded into 96-well plates at a density of 1 × 10^4^ cells per well and treated with IL-13 130R or IL-13 130Q (1–100 ng/mL) for 24 h. Subsequently, 10 μL of CCK-8 solution (Beyotime, Jiangsu, China) was added to each well, and the cells were incubated at 37 °C for 1 h. Absorbance at 450 nm was measured using an enzyme immunoassay analyzer (Bio-Rad, Hercules, CA, USA). Because CCK-8 reflects cellular dehydrogenase activity, the readout was interpreted as CCK-8-derived proliferative/metabolic activity rather than as a direct measurement of DNA synthesis or cell division.

### Cell migration assay

Migration of hBSMCs was assessed using a standard Boyden chamber assay with an 8-μm-pore polycarbonate membrane (Costar, Corning, NY, USA). Briefly, hBSMCs were serum-deprived for 24 h, detached with trypsin–ethylenediaminetetraacetic acid (Invitrogen Canada Inc., Burlington, ON, Canada), washed, and resuspended in SMCM at a density of 5 × 10^4^ cells/mL. The cell suspension was added to the upper chamber, whereas the lower chamber contained culture medium supplemented with IL-13 130R or IL-13 130Q at concentrations ranging from 1 to 100 ng/mL. PBS without IL-13 served as the negative control. After 24 h of incubation, cells that had migrated to the underside of the membrane were counted under a phase-contrast microscope (OPTIPHOT, Nikon, Japan) at ×400 magnification. Five random fields were counted for each chamber.

### Determination of intracellular calcium concentration

Following pretreatment with IL-13 130R or IL-13 130Q (1–100 ng/mL) for 24 h, hBSMCs were incubated in SMCM containing 5 μM Fluo-3/AM (Beyotime, Jiangsu, China) under light-protected conditions for 1 h, followed by an additional 30 min of incubation in extracellular saline. Cells were detached with trypsin, gently pipetted to obtain a single-cell suspension, and centrifuged at 1,000 rpm for 5 min at 2–8 °C. The cell pellet was washed with ice-cold PBS and resuspended in 500 μL PBS containing 10 μM ACh, as previously described (Adapala et al., 2011). Relative median Fluo-3/AM fluorescence intensity, reflecting the ACh-induced intracellular calcium response, was measured by flow cytometry (Bio-Rad, Hercules, CA, USA).

### ELISA for IL-4 and IL-5

After stimulation with various concentrations of IL-13 130R or IL-13 130Q for 24 h, culture supernatants from hBSMCs were collected for cytokine analysis. According to the instructions of the manufacturer for the IL-4 and IL-5 ELISA kits (RayBiotech, Peachtree Corners, GA, USA), 100 μL of each sample was added to pre-coated wells and incubated at room temperature for 2.5 h. The wells were then washed three times for 5 min each, and blank wells served as negative controls. Horseradish peroxidase (HRP)-conjugated detection antibody was added and incubated for 1 h at room temperature, followed by incubation with streptavidin solution under gentle shaking for 45 min. Subsequently, 100 μL of tetramethylbenzidine substrate was added, and the plates were incubated in the dark for 30 min. The reaction was terminated by adding 100 μL of stop solution, and absorbance at 450 nm was measured within 30 min against the blank control. Concentrations of IL-4 and IL-5 in hBSMC culture supernatants were calculated from standard curves.

### Histopathological assessment

The left lung from each mouse was excised, embedded in paraffin, and sectioned into 4-μm slices. Lung sections were stained with hematoxylin and eosin for histopathological evaluation. Bronchial assessment was performed in triplicate in a blinded manner using the following criteria: bronchi with a circular shape, a diameter of 200–400 μm, and a major-to-minor axis ratio of < 1.2 (Hu et al., 2019). In addition, the bronchial basement membrane perimeter (Pbm), airway smooth muscle area (Wam), total bronchial wall area (Wat), and inner airway area (Wai) were measured using the Image-Pro Plus 6.0 medical image analysis system (Media Cybernetics, Rockville, MD, USA). All measurements were normalized to Pbm.

### Western blot

Cells were lysed in radioimmunoprecipitation assay buffer containing phenylmethylsulfonyl fluoride and phosphatase inhibitors and centrifuged at 12,000 rpm for 5 min at 4 °C. The supernatants were collected and stored at −20 °C. Protein concentrations were determined using the bicinchoninic acid assay. Protein samples were denatured in 5× loading buffer at 100 °C for 5 min, separated on 12% or 6% sodium dodecyl sulfate-polyacrylamide gel electrophoresis gels, and transferred onto polyvinylidene fluoride membranes. After blocking with 5% non-fat milk, the membranes were incubated overnight at 4 °C with primary antibodies against total Janus kinase 2 (T-JAK2; Abcam, Cambridge, UK; 1:1,000), phosphorylated JAK2 (p-JAK2; Abcam, Cambridge, UK; 1:1,000), total signal transducer and activator of transcription 6 (T-STAT6; Abcam, Cambridge, UK; 1:1,000), and phosphorylated STAT6 (p-STAT6; Abcam, Cambridge, UK; 1:1,000). Membranes were then incubated with HRP-conjugated secondary antibodies (1:5,000) for 2 h at room temperature. Protein bands were visualized using enhanced chemiluminescence reagents and quantified with Image Lab software, using tubulin as the loading control.

### Protein modeling and molecular dynamics simulation

The amino acid sequence and accession number of IL-13 were obtained from the UniProt database (http://www.uniprot.org/). The Project HOPE database (http://www.cmbi.ru.nl/hope) was used to predict the structural effects of the IL-13 R130Q mutation. Structural modeling of the IL-13 R130Q variant was then performed using the SWISS-MODEL (http://swissmodel.expasy.org/). The corresponding Protein Data Bank (PDB) file was generated using http://swissmodel.expasy.org/interactive/VFDA89/. Subsequently, SPDBV-4.04-PC was used to construct three-dimensional (3D) structures of wild-type IL-13 and the IL-13 R130Q variant based on the protein sequence template from the PDB file. A 3D structural region encompassing amino acid residues adjacent to the mutation site was generated to evaluate local structural changes.

The crystal structure of the IL-13/IL-13 receptor alpha 2 (IL-13Rα2) complex (PDB ID: 3LB6) was retrieved from the Protein Data Bank. The R130Q mutant complex was generated using AlphaFold3 (Abramson et al., 2024), and the AlphaFold3-derived structure was used for all production molecular dynamics simulations. Three independent 100-ns simulations were performed for each system using GROMACS 2025.4 with the AMBER ff19SB force field in an explicit solvent environment. The protein complexes were solvated in an explicit optimal point charge water box containing physiological ion concentrations. Energy minimization was performed using the steepest-descent algorithm, followed by 0.5 ns of equilibration with gradual heating from 0 K to 298 K. Production simulations were conducted under constant particle-number, pressure, and temperature conditions, maintaining a temperature of 298 K with the V-rescale thermostat and a pressure of 1 bar with the C-rescale barostat. Long-range electrostatic interactions were calculated using the particle mesh Ewald method with a 10-Å cutoff. Hydrogen bonds were constrained using the LINCS algorithm with a 2-fs integration time step. Trajectory analyses included root mean square deviation (RMSD), root mean square fluctuation (RMSF), radius of gyration (Rg), solvent-accessible surface area (SASA), and hydrogen-bond analyses. Binding free energies were estimated from the final 10 ns of each independent trajectory using the molecular mechanics/generalized Born surface area (MM/GBSA) method implemented in gmx_MMPBSA (Valdes-Tresanco et al., 2021), with entropy contributions omitted. MM/GBSA values were used for relative ranking of the two complexes rather than as absolute binding free energies. Interaction diagrams were generated using LigPlot+ and PyMOL.

### Statistical analysis

Biological data are presented as the mean ± standard error of the mean, whereas molecular dynamics data are presented as the mean ± standard deviation across three independent simulations. Comparisons between two groups were performed using the unpaired Student’s t-test. Comparisons among multiple groups were performed using one-way analysis of variance (ANOVA) followed by Bonferroni multiple-comparison tests. Dose-by-variant experiments were analyzed using two-way ANOVA followed by Bonferroni multiple-comparison tests. Molecular dynamics parameters and relative MM/GBSA binding free energies were compared using the Mann–Whitney U test. All statistical analyses were performed using SPSS version 21.0 (SPSS Inc., Chicago, IL, USA). A two-sided P value < 0.05 was considered statistically significant.

## Results

### IL-13 130Q aggravated airway remodeling in asthmatic mice

**Figure 2:**
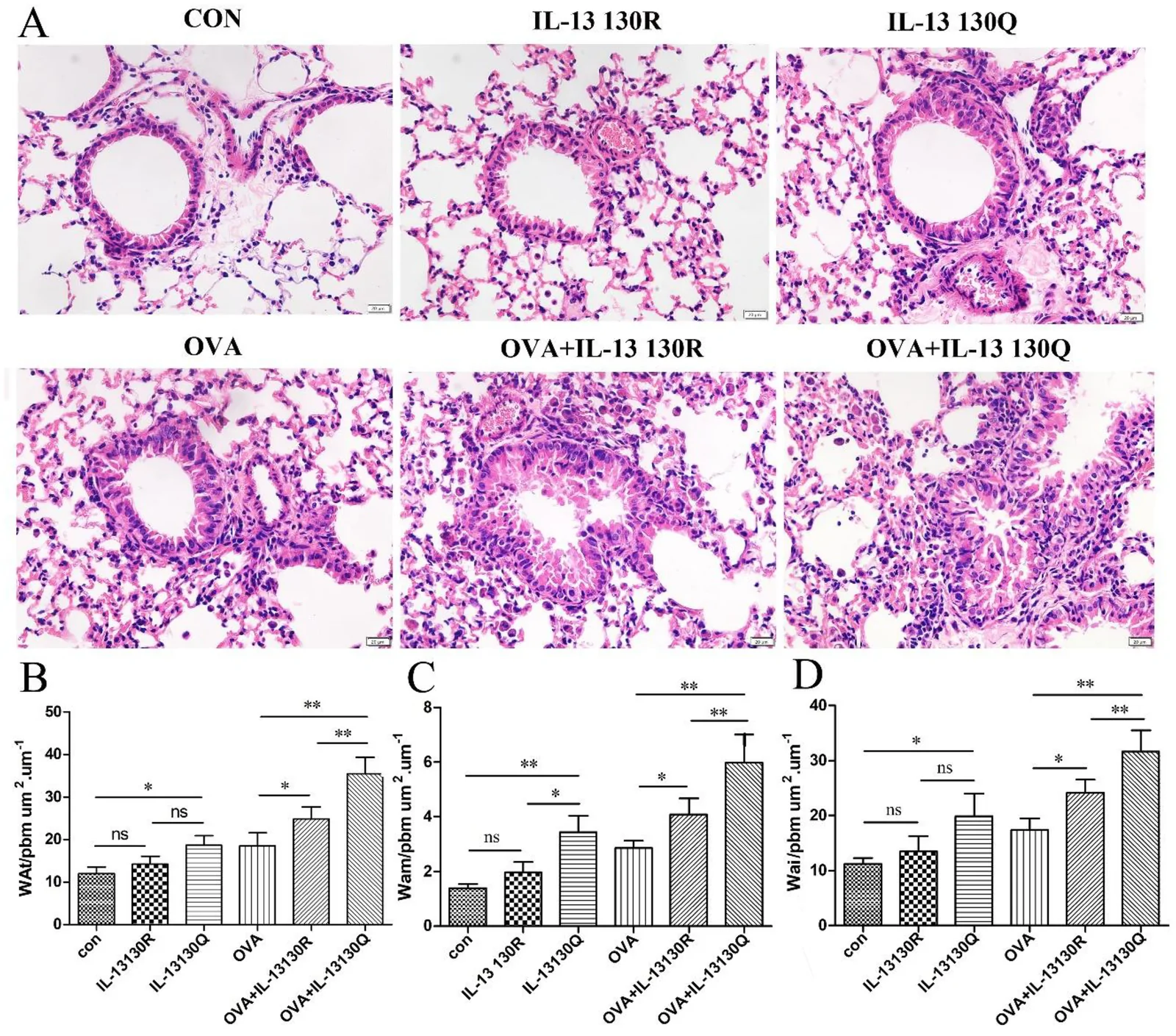
Effects of IL-13 130R and IL-13 130Q on airway remodeling in severe asthma. H&E staining was performed in rodent pulmonary sections to display histopathological characteristics **(A)** and indicators relating to asthmatic airway remodeling: Wat/Pbm **(B)**, Wam/Pbm **(C)**, Wai/Pbm **(D)**. Data are shown as mean ± SEM (n = 8 for each group). *P < 0.05, **P < 0.01. Pbm, basement membrane perimeter; Wai, inner airway area; Wat, total bronchial wall area; Wam, smooth-muscle area; H&E, hematoxylin and eosin; SEM, standard error of the mean; IL, interleukin, OVA, ovalbumin.

Hematoxylin and eosin staining of mouse lung tissue showed mild airway changes in mice treated with IL-13 130R or IL-13 130Q compared with the control group, including airway wall thickening, luminal narrowing, increased airway smooth muscle (ASM) mass, and inflammatory cell infiltration (**Figure 2A**). In OVA-sensitized mice, pretreatment with IL-13 130R or IL-13 130Q further exacerbated the histopathological features of airway remodeling compared with the OVA group alone (**Figure 2A**). This was reflected by significant increases in total bronchial wall area normalized to the basement membrane perimeter (Wat/Pbm; **Figure 2B**), airway smooth-muscle area normalized to Pbm (Wam/Pbm; **Figure 2C**), and inner airway area normalized to Pbm (Wai/Pbm; **Figure 2D**). Histopathological abnormalities were more severe in OVA-sensitized and challenged mice treated with IL-13 130Q than in those treated with IL-13 130R, indicating a stronger potentiating effect of the 130Q variant in the allergic airway environment.

### IL-13 130Q potentiated pulmonary inflammatory responses in OVA-induced asthma

**Figure 3:**
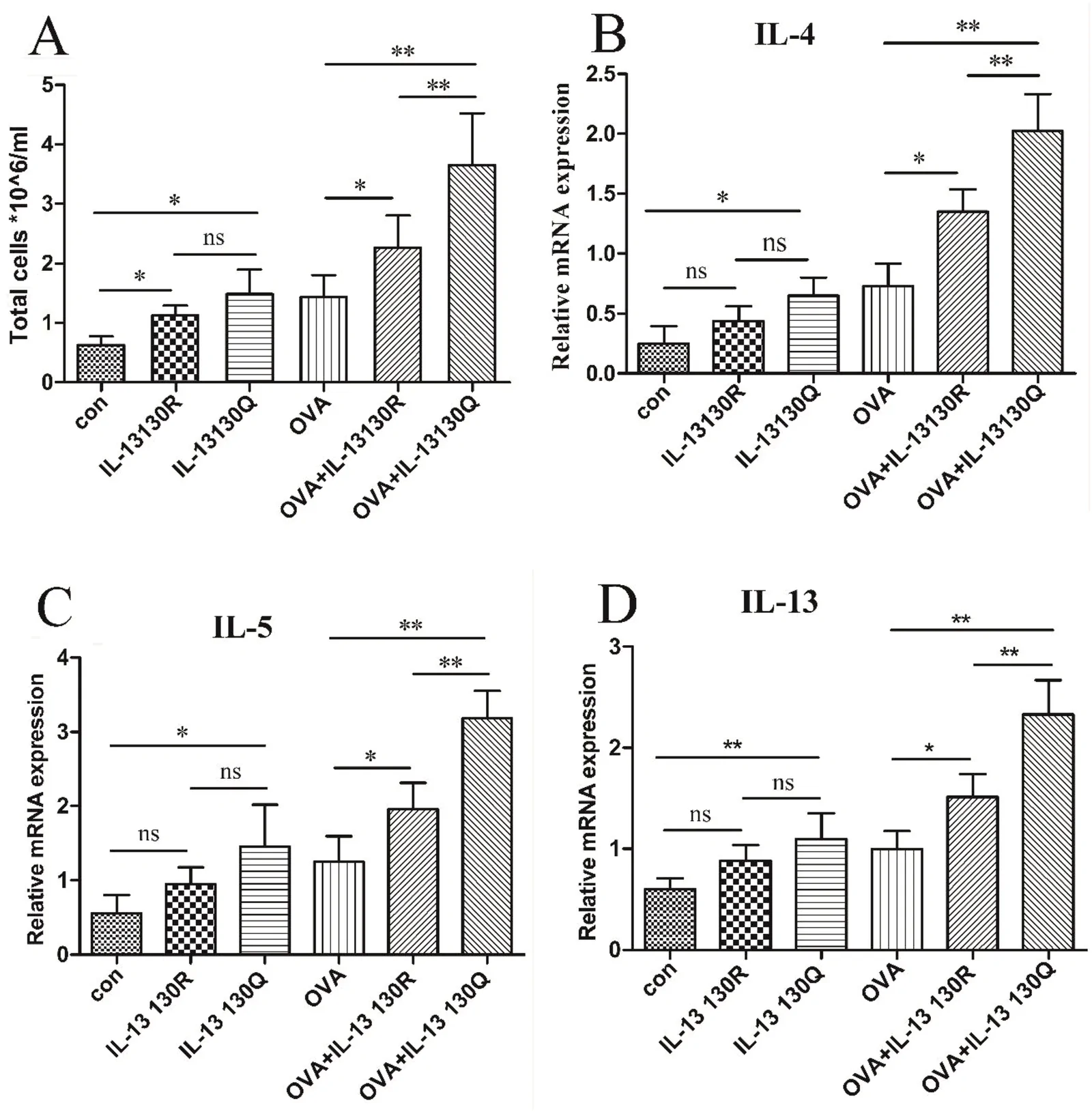
Effects of IL-13 130R and IL-13 130Q on pulmonary inflammatory responses. **(A)** Total cell counts in bronchoalveolar lavage fluid (BALF; n = 8 per group); differential leukocyte counting was not performed. **(B–D)** Relative mRNA expression levels of IL-4 **(B)**, IL-5 **(C)**, and IL-13 **(D)** in lung tissue (n = 8 per group). Data are presented as the mean ± SEM. *P < 0.05, **P < 0.01. IL, interleukin; OVA, ovalbumin; SEM, standard error of the mean.

The effects of IL-13 130R and IL-13 130Q on pulmonary inflammation were evaluated by measuring total BALF cell counts and relative mRNA expression levels of IL-4, IL-5, and IL-13 in lung tissue. Although total BALF cell counts and cytokine mRNA expression tended to increase following IL-13 administration in mice not exposed to OVA, no significant differences were observed between the IL-13 130R and IL-13 130Q groups (**Figures 3A–D**). In contrast, mice in the OVA + IL-13 130Q group exhibited significantly higher total BALF cell counts and lung cytokine mRNA expression than mice in the OVA + IL-13 130R group (**Figures 3A–D**). Differential leukocyte counts were not performed; therefore, the individual contributions of eosinophils, neutrophils, lymphocytes, mast cells, basophils, and other cell populations could not be determined. These findings indicate that IL-13 130Q more strongly potentiates the pulmonary inflammatory response in the OVA-induced allergic environment.

### IL-13 130Q increased CCK-8-derived proliferative/metabolic activity in hBSMCs

**Figure 4:**
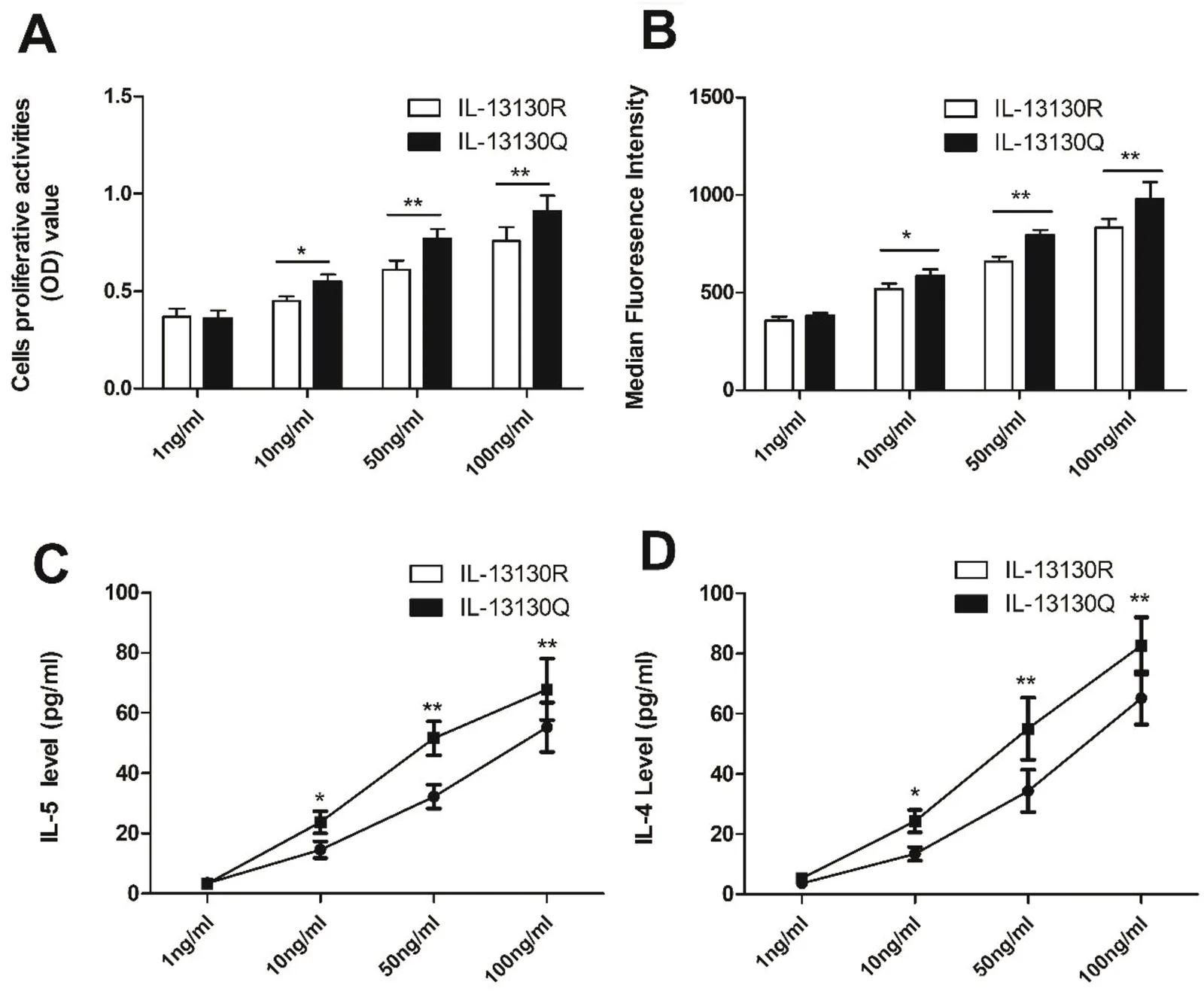
Effects of IL-13 130R and IL-13 130Q on human bronchial smooth muscle cell (hBSMC) phenotypes (n = 4 per group). **(A)** CCK-8-derived proliferative/metabolic activity. **(B)** Representative Fluo-3/AM flow-cytometry histograms and median fluorescence intensity quantification of the acetylcholine-induced intracellular calcium response. **(C, D)** IL-5 **(C)** and IL-4 **(D)** concentrations in culture supernatants. Data are presented as the mean ± SEM. *P < 0.05, **P < 0.01. IL, interleukin; SEM, standard error of the mean.

CCK-8-derived proliferative/metabolic activity was assessed after treatment with IL-13 130R or IL-13 130Q. Both variants produced concentration-dependent increases in the CCK-8 readout. IL-13 130Q produced a significantly greater increase than IL-13 130R at concentrations of 10, 50, and 100 ng/mL (P < 0.05; **Figure 4A**), whereas no significant difference was observed at 1 ng/mL. Because CCK-8 does not directly measure DNA synthesis or cell division, these data indicate increased proliferative/metabolic activity rather than definitive evidence of cell proliferation.

### IL-13 130Q enhanced the ACh-induced intracellular calcium response in hBSMCs

The effects of IL-13 130R and IL-13 130Q on the ACh-induced intracellular calcium response were evaluated by flow cytometry using Fluo-3/AM. After 24 h of treatment with increasing concentrations of either IL-13 variant followed by stimulation with 10 μM ACh, IL-13 130Q induced significantly greater increases in median Fluo-3/AM fluorescence intensity than IL-13 130R at concentrations of 10–100 ng/mL (P < 0.05; **Figure 4B**). These measurements reflect intracellular calcium signaling and do not directly quantify hBSMC contractile force.

### IL-13 130Q increased IL-4 and IL-5 concentrations in hBSMC culture supernatants

ELISA was performed on hBSMC culture supernatants after 24 h of stimulation with IL-13 130R or IL-13 130Q. Both variants were associated with concentration-dependent increases in IL-4 and IL-5 concentrations over the range of 10–100 ng/mL. Compared with IL-13 130R, IL-13 130Q produced significantly higher IL-4 and IL-5 concentrations at 10, 50, and 100 ng/mL (P < 0.05; **Figures 4C, D**), with the greatest differences observed at 50 and 100 ng/mL (P < 0.01). Because IL-13-induced IL-4 and IL-5 production by hBSMCs has not been independently established, these unexpected ELISA findings should be regarded as preliminary and condition-dependent.

### IL-13 130Q promoted hBSMC migration

**Figure 5:**
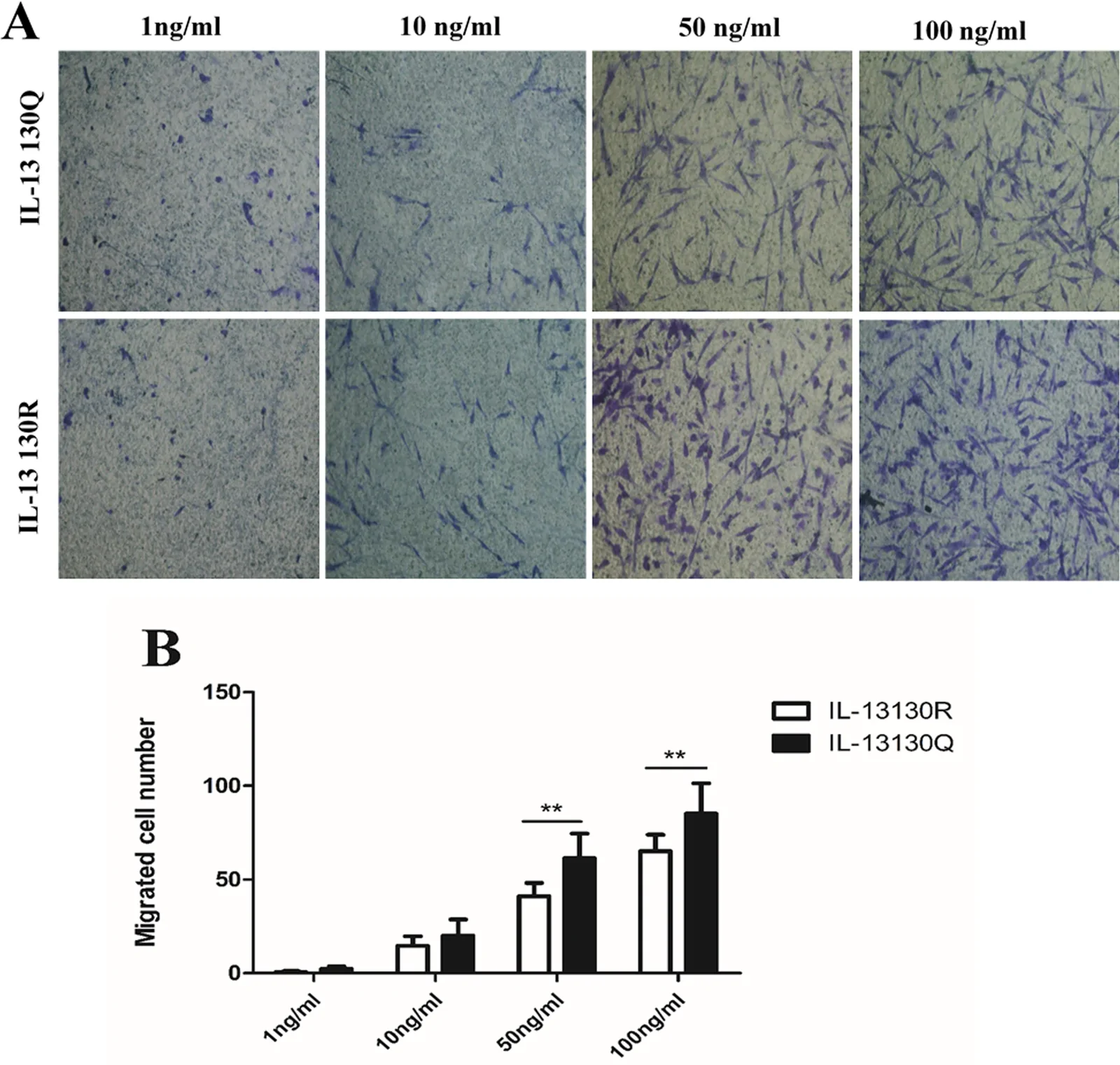
Effects of IL-13 130R and IL-13 130Q on hBSMC migration. **(A)** Representative images of hBSMCs that migrated through the Boyden chamber membrane after 24 h of treatment (×400 magnification). **(B)** Quantification of migrated cells (n = 4 per group). Data are presented as the mean ± SEM. *P < 0.05, **P < 0.01. hBSMCs, human bronchial smooth muscle cells; IL, interleukin; SEM, standard error of the mean.

A Boyden chamber assay was used to evaluate the relative migratory capacity of hBSMCs after treatment with IL-13 130R or IL-13 130Q. A greater number of hBSMCs migrated through the polycarbonate membrane in response to IL-13 130Q than to IL-13 130R (**Figure 5A**), with significant differences at 50 and 100 ng/mL (P < 0.01; **Figure 5B**). Thus, IL-13 130Q induced a stronger concentration-dependent migratory phenotype *in vitro*. The 24-h assay does not directly demonstrate hBSMC migration *in vivo* or establish a causal contribution to subepithelial fibrosis.

### R130Q was associated with reduced predicted IL-13/IL-13Rα2 binding stability despite preserved overall structural integrity

**Figure 6:**
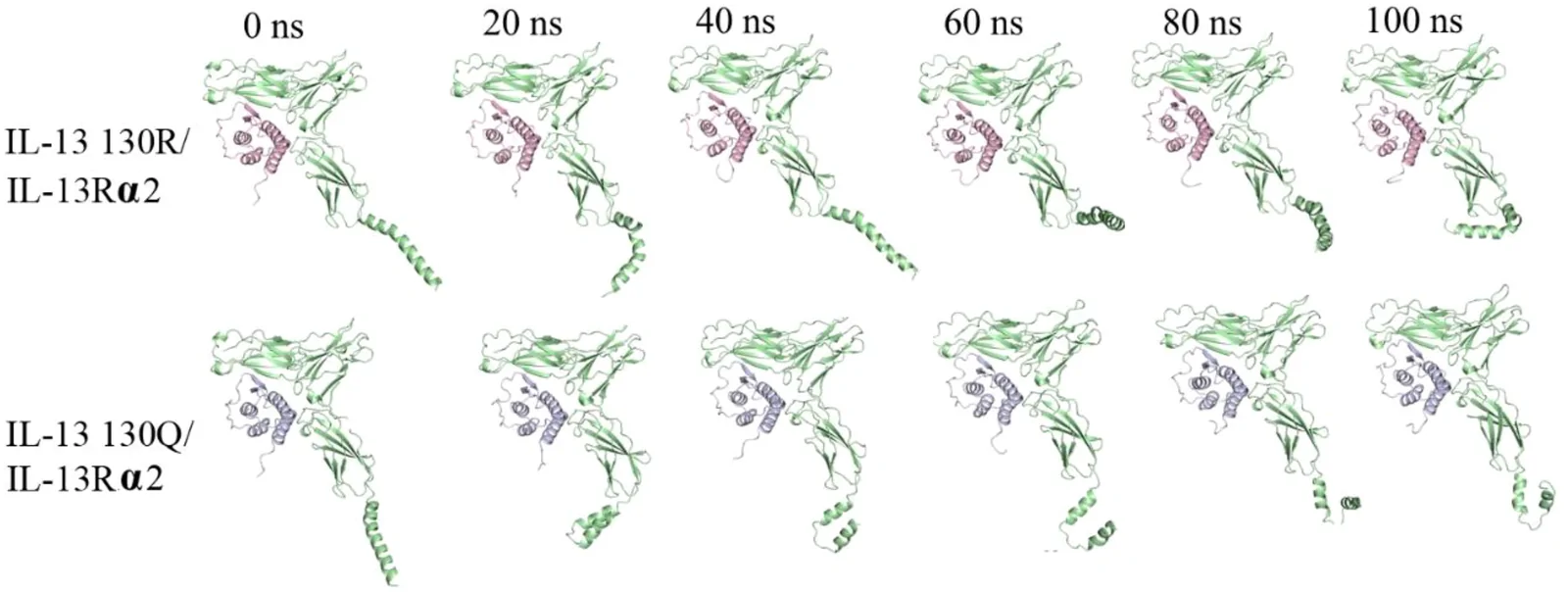
Structural snapshots of the IL-13/IL-13 receptor α2 complex at different time points during molecular dynamics simulation. IL, interleukin.

The three-dimensional structures of the wild-type IL-13 130R/IL-13Rα2 and IL-13 130Q/IL-13Rα2 complexes were generated from the crystal structure (PDB ID: 3LB6) and the AlphaFold3 prediction. Structural comparison showed similar initial binding modes and interfaces, with no obvious conformational rearrangements in the static models (**Figure 6**). The R130Q substitution therefore did not markedly alter the initial overall folding architecture, and molecular dynamics simulations were used to assess local dynamics and predicted binding stability.

**Figure 7:**
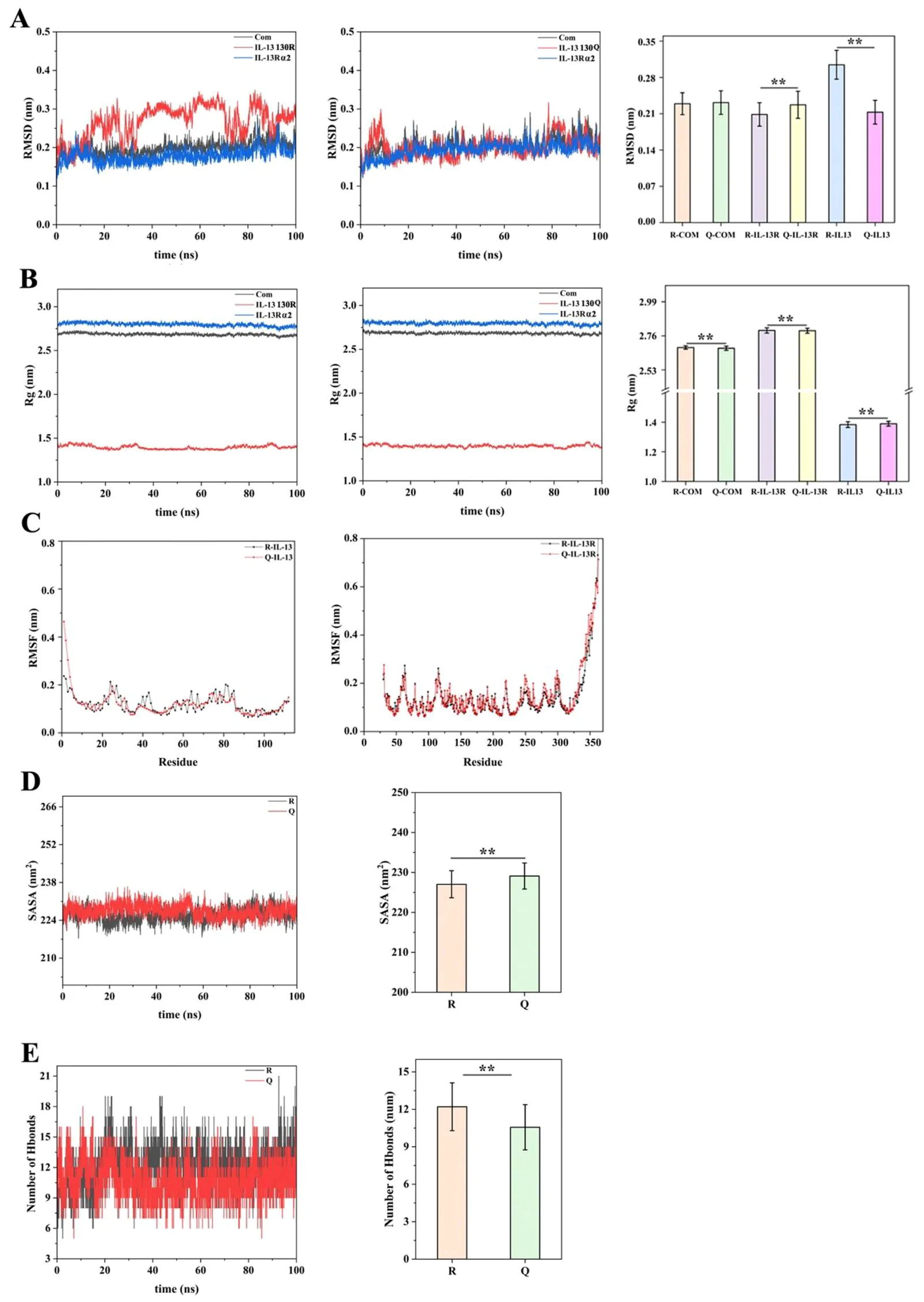
Molecular dynamics analyses of the IL-13 130R/IL-13Rα2 and IL-13 130Q/IL-13Rα2 complexes. **(A)** Root mean square deviation (RMSD) and **(B)** radius of gyration (Rg) during the 100-ns simulations. In panels A and B, the left graphs represent the IL-13 130R/IL-13Rα2 complex and the right graphs represent the IL-13 130Q/IL-13Rα2 complex; curves indicate the whole complex, IL-13, and IL-13Rα2, respectively. **(C)** Root mean square fluctuation (RMSF) of IL-13 (left) and IL-13Rα2 (right) in the two complexes. **(D)** Solvent-accessible surface area (SASA) and **(E)** number of hydrogen bonds during the simulations (n = 3 independent simulations per group). R and Q denote the complexes containing IL-13 130R and IL-13 130Q, respectively. Data are presented as the mean ± SD. **P < 0.01. SD, standard deviation.

**Figure 8:**
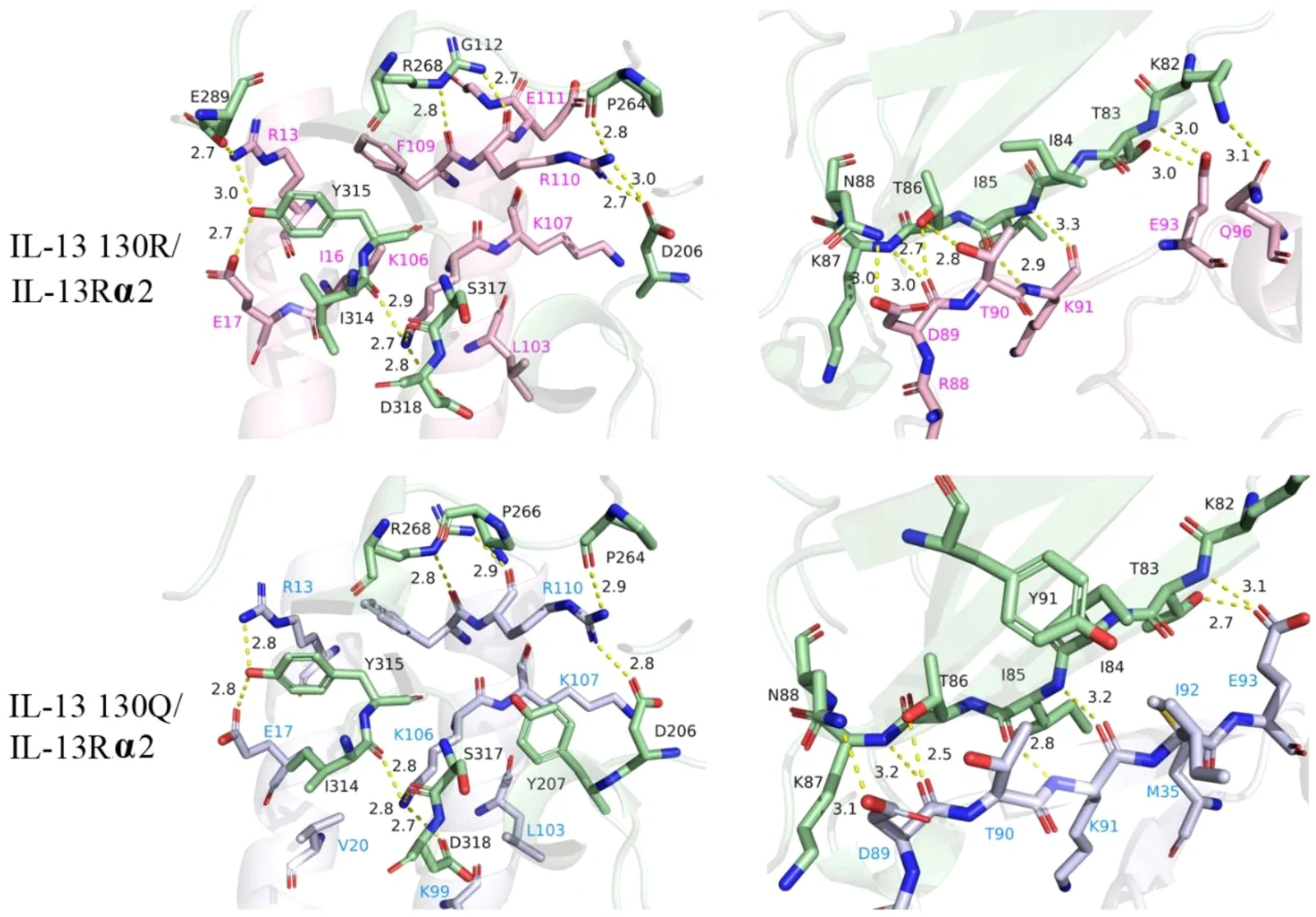
Close-up views of the IL-13/IL-13Rα2 binding interface. The upper and lower rows show the IL-13 130R/IL-13Rα2 and IL-13 130Q/IL-13Rα2 complexes, respectively; the left and right panels show two regions of the binding interface. IL, interleukin.

**Figure 9:**
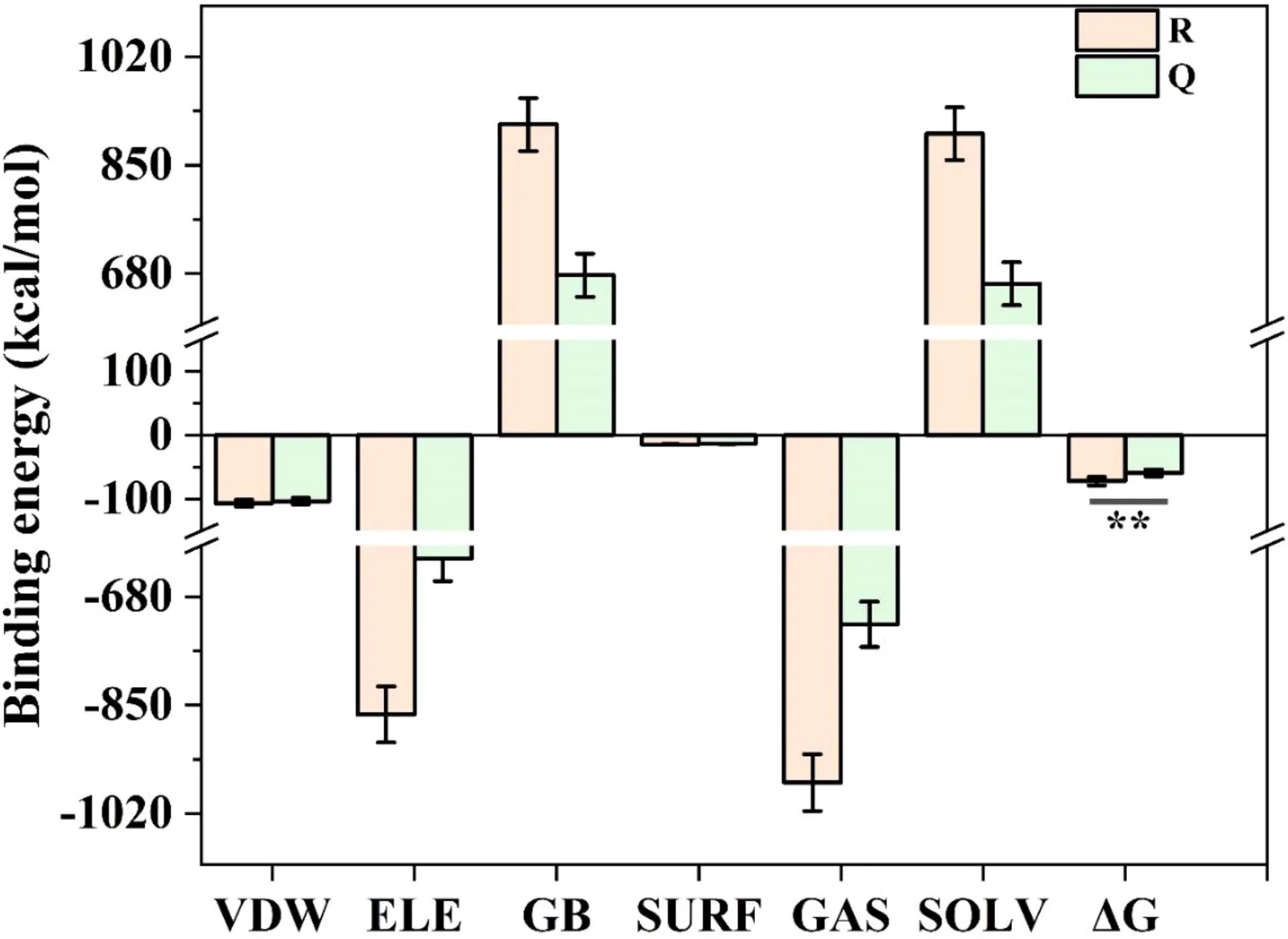
Relative MM/GBSA binding free-energy components of the IL-13/IL-13Rα2 complexes (n = 3 independent simulations per group). R and Q denote complexes containing IL-13 130R and IL-13 130Q, respectively. VDW, van der Waals energy; ELE, electrostatic energy; GB, polar solvation energy; SURF, nonpolar solvation energy; GAS, gas-phase energy (GAS = VDW + ELE); SOLV, solvation energy (SOLV = GB + SURF); ΔG, relative binding free energy (ΔG = GAS + SOLV). Data are presented as the mean ± SD. **P < 0.01. MM/GBSA values were used for relative comparison and should not be interpreted as absolute binding affinities.

Across three independent 100-ns simulations, both systems reached equilibrium after approximately 20 ns, and the RMSD and Rg profiles indicated preservation of overall structural integrity (**Figures 7A, B**). RMSF analysis showed that, within the IL-13/IL-13Rα2 complexes, IL-13 130R exhibited lower residue-level fluctuations than IL-13 130Q in interface-associated regions comprising residues 13–17 and 90–110 (**Figure 7C**), indicating greater local conformational rigidity rather than a generalized difference in overall binding flexibility. The complexes also differed in SASA (P < 0.01; **Figure 7D**). The IL-13 130R/IL-13Rα2 complex maintained an average of 12.20 ± 1.92 hydrogen bonds, whereas the IL-13 130Q/IL-13Rα2 complex maintained 10.56 ± 1.81 (P < 0.01; **Figure 7E**). At the interaction interface, contacts involving R110 and receptor residues D206 and R268 were attenuated, and side-chain hydrogen bonds between IL-13 residues Q96 and T90 and IL-13Rα2 residues K82 and T86 were lost in the variant complex (**Figure 8**). Relative MM/GBSA calculations based on the final 10 ns of each independent trajectory yielded a more favorable value for the IL-13 130R complex (−72.1 ± 6.7 kcal/mol) than for the IL-13 130Q complex (−59.3 ± 5.3 kcal/mol; P < 0.01; **Figure 9**). Because MM/GBSA provides relative rather than absolute affinity estimates, these results support reduced predicted binding stability of IL-13 130Q to IL-13Rα2 but do not constitute a direct receptor-binding measurement.

### IL-13 130Q induced greater JAK2/STAT6 phosphorylation in hBSMCs

**Figure 10:**
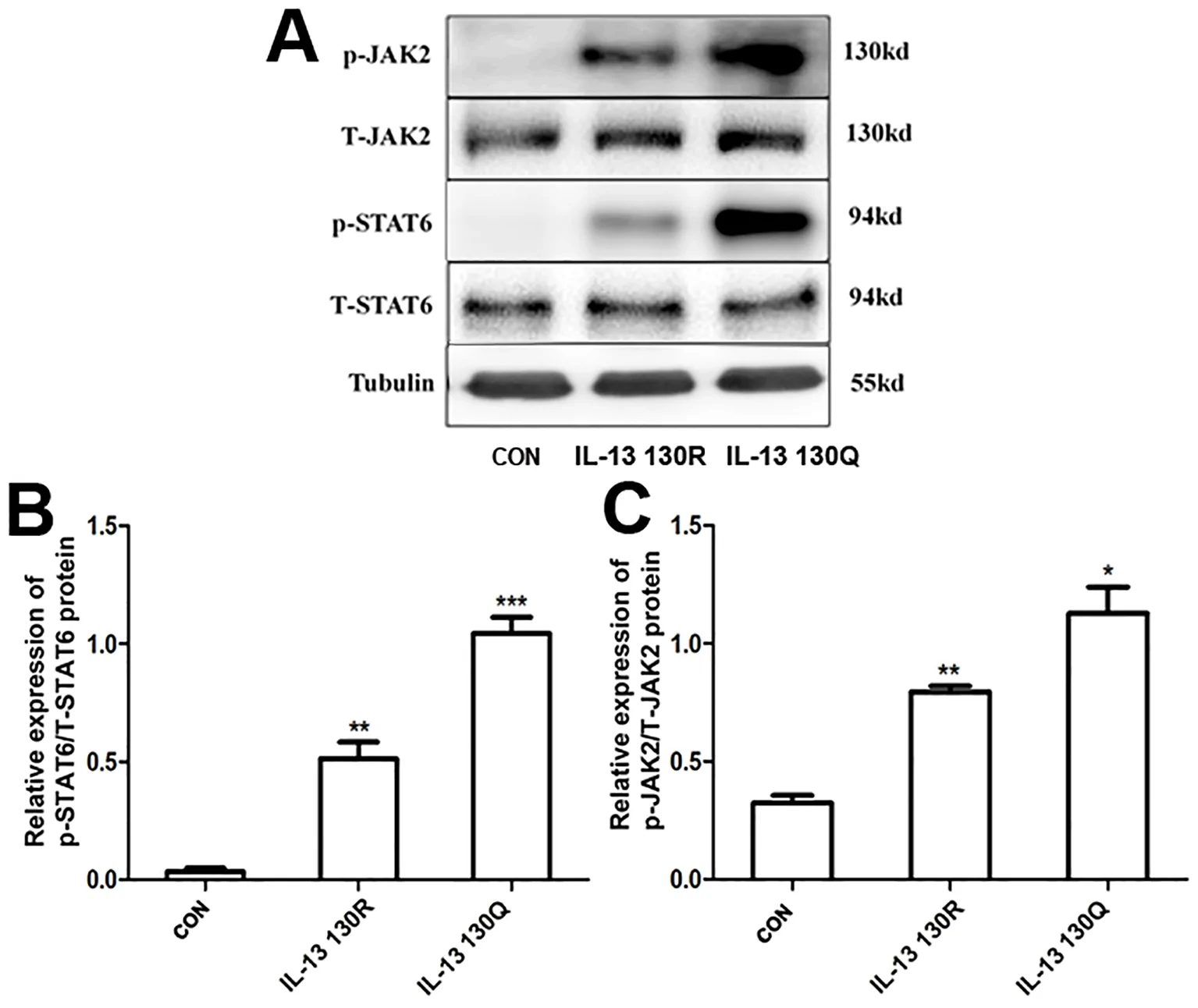
Effects of IL-13 130R and IL-13 130Q on JAK2/STAT6 signaling in hBSMCs. **(A)** Representative Western blots of phosphorylated JAK2 (p-JAK2), total JAK2, phosphorylated STAT6 (p-STAT6), total STAT6, and tubulin. **(B, C)** Quantification of p-STAT6/total STAT6 **(B)** and p-JAK2/total JAK2 **(C)** (n = 4 per group). Data are presented as the mean ± SEM. Statistical significance is indicated for the comparisons shown: *P < 0.05, **P < 0.01, ***P < 0.001. Con, control; hBSMCs, human bronchial smooth muscle cells; JAK, Janus kinase; STAT, signal transducer and activator of transcription; SEM, standard error of the mean.

After 24 h of treatment, Western blot analysis showed increased p-JAK2 and p-STAT6 levels in hBSMCs exposed to either IL-13 variant compared with the control group (**Figure 10**). Both p-JAK2 and p-STAT6 levels were higher following IL-13 130Q treatment than following IL-13 130R treatment. These findings indicate that IL-13 130Q elicits a stronger downstream JAK2/STAT6 signaling response in hBSMCs. The phosphorylation data are functional signaling readouts and do not directly demonstrate altered binding of IL-13 130Q to IL-13Rα1 or IL-13Rα2.

## Discussion

During the pathogenesis of asthma, ASM cells undergo dynamic phenotypic modulation. This phenotypic switching is characterized by several key features: (i) ASM hypertrophy and abnormal hyperplasia (James et al., 2012; Pham et al., 2021); (ii) enhanced contractile responsiveness, which contributes to airway hyperresponsiveness (Pham et al., 2021; Khalfaoui and Pabelick, 2023); (iii) acquisition of a pronounced synthetic phenotype, whereby ASM cells become an important source of extracellular matrix components, including type I collagen and fibronectin, during airway remodeling (Liu et al., 2017; Nedeva et al., 2023; Savin et al., 2023); (iv) increased secretion of inflammatory mediators and chemokines, including IL-6, IL-8, chemokine (C-C motif) ligand 19, and eotaxin (Xia et al., 2013); and (v) augmented migratory capacity (Kardas et al., 2020; Janulaityte et al., 2021).

Enhanced ASM migration may contribute to the accumulation or redistribution of smooth-muscle or myofibroblast-like cells within the remodeled airway wall and thereby participate in airway narrowing and subepithelial remodeling (Kardas et al., 2020; Janulaityte et al., 2021). However, migration measured in a short-term Boyden chamber assay represents an *in vitro* phenotype and should not be interpreted as direct evidence of cell movement from the smooth-muscle layer to the subepithelial compartment *in vivo*. More broadly, ASM phenotypic transformation reflects a shift from a predominantly contractile state toward synthetic, proliferative, secretory, or migratory states in response to inflammatory and mechanical stimuli (Qiu et al., 2019). These changes are closely associated with airway remodeling and poor disease control in severe asthma (Pepe et al., 2005; Joseph and Tatler, 2022; Tiotiu et al., 2025).

Accumulating evidence indicates that IL-13 is a key inflammatory cytokine involved in asthma pathogenesis. IL-13 regulates multiple ASM phenotypes and functions, including proliferation, migration, contractility, and inflammatory cytokine secretion (Wright et al., 2013; Xiang et al., 2022). Through these effects, it promotes airway remodeling and contributes to disease progression. The IL-13 R130Q variant is a common single-nucleotide polymorphism in the IL-13 gene. This variant has been associated with elevated serum immunoglobulin E levels (Graves et al., 2000), increased peripheral blood eosinophil counts (Palikhe et al., 2010), and greater susceptibility to and severity of asthma (Nagashima et al., 2011; Mei and Qu, 2017; Omraninava et al., 2022). In a large-scale clinical screening study of Han Chinese children, our group previously found that the IL-13 R130Q variant was significantly enriched among patients with asthma (Hua et al., 2016).

Our previous studies investigated the mechanisms by which IL-13 130Q contributes to severe asthma from the perspectives of pulmonary inflammation and airway hyperresponsiveness. These studies showed that IL-13 130Q may exert its effects by markedly increasing eotaxin release from ASM cells and inducing modest but significant upregulation of smooth muscle-specific markers, including alpha-smooth muscle actin, smooth muscle myosin heavy chain, calreticulin, and the Fc fragment of immunoglobulin E receptor I α-chain (He et al., 2013). However, the role of this variant in ASM phenotypic modulation and subsequent airway remodeling remains incompletely understood.

Our *in vitro* experiments showed that IL-13 130Q induced greater CCK-8-derived proliferative/metabolic activity, ACh-induced intracellular calcium responses, migration, and IL-4 and IL-5 concentrations in hBSMC culture supernatants than IL-13 130R. Airway smooth muscle was traditionally viewed primarily as a contractile tissue, but it is now recognized as an immunomodulatory structural compartment with substantial phenotypic plasticity and secretory capacity (Xia et al., 2013; Camoretti-Mercado and Lockey, 2021). Limited but direct evidence indicates that human airway smooth muscle cells can release IL-4 and IL-5 under specific allergic activation conditions. Gounni et al. demonstrated release of IL-4, IL-5, IL-13, and eotaxin after IgE sensitization and FcϵRI cross-linking (Gounni et al., 2005), whereas Hakonarson et al. reported increased IL-5 mRNA expression and protein release after sensitization with serum from patients with atopic asthma (Hakonarson et al., 1999). Nevertheless, these studies did not establish IL-13-induced production of IL-4 or IL-5. Therefore, the present ELISA findings should be interpreted as unexpected, preliminary, and condition-dependent observations requiring confirmation in additional donors and by orthogonal molecular and protein assays. Similarly, the Fluo-3/AM assay reflects intracellular calcium signaling rather than directly measured contractile force, and the CCK-8 assay does not directly quantify DNA synthesis or cell division.

To investigate the molecular basis underlying the differential functional activities of the two variants, we separately evaluated their interactions with IL-13Rα2 using structural modeling and molecular dynamics simulations and assessed JAK2/STAT6 phosphorylation as a downstream signaling readout. (Roger et al., 2021) Although both IL-13/IL-13Rα2 complexes preserved their overall structural integrity, RMSF analysis showed lower residue-level fluctuations for IL-13 130R than for IL-13 130Q in interface-associated regions, indicating greater local conformational rigidity rather than a global difference in ‘binding flexibility.’ The IL-13 130Q/IL-13Rα2 complex also exhibited fewer interfacial hydrogen bonds, loss of selected contacts involving IL-13 Q96/T90 and receptor K82/T86, and a less favorable relative MM/GBSA binding free energy. These computational findings suggest reduced predicted stability of the IL-13 130Q/IL-13Rα2 interaction. However, MM/GBSA estimates are relative, and the simulations did not directly measure receptor-binding affinity.

IL-13 exerts its biological effects through receptor complexes involving IL-13Rα1, whereas IL-13Rα2 can function as a high-affinity decoy receptor that sequesters local IL-13 and limits signaling (Zheng et al., 2008). Based on the computationally predicted reduction in IL-13Rα2 binding stability, we hypothesize that IL-13 130Q may undergo less efficient sequestration by the decoy receptor, potentially leaving more free ligand available for signaling through the functional IL-13 receptor complex. Consistent with this proposed model, IL-13 130Q induced greater JAK2 and STAT6 phosphorylation than IL-13 130R in hBSMCs. Nevertheless, phosphorylation does not directly demonstrate reduced IL-13Rα2 binding or increased IL-13Rα1 binding. The current study did not simulate or measure the IL-13/IL-13Rα1 interaction, confirm receptor abundance in the experimental hBSMCs, or functionally perturb IL-13Rα2. Thus, altered direct affinity for IL-13Rα1 and other mechanisms cannot be excluded, and the decoy-receptor model should be regarded as one plausible explanation rather than a validated causal pathway. IL-13 may also engage alternative signaling cascades, including ERK1/2–AP-1, PI3K/Akt, and MAPK pathways, which could contribute to the observed phenotype (Shankar et al., 2022).

The *in vivo* effects of IL-13 130Q were strongly dependent on the underlying allergic context. Administration of either variant to mice not exposed to OVA produced only limited airway changes, whereas IL-13 130Q markedly aggravated airway wall thickening, luminal narrowing, ASM expansion, total BALF cell accumulation, and type 2 cytokine transcription in OVA-sensitized and intranasally challenged mice compared with IL-13 130R. These findings suggest that IL-13 130Q acts primarily as a context-dependent potentiating variant in an established type 2 inflammatory environment rather than independently producing a distinct asthma phenotype. The OVA-dependent *in vivo* findings do not conflict with the OVA-independent *in vitro* effects: the animal model contains complex epithelial, immune, stromal, and mechanical interactions, whereas the simplified cell system directly tests the intrinsic responsiveness of hBSMCs to the two IL-13 variants. Because **Figure 3A** represents total BALF cell counts, the specific leukocyte populations responsible for the increase remain unknown. Moreover, the study did not test simultaneous administration of IL-13 130R and IL-13 130Q and therefore cannot determine whether the variants have additive or synergistic effects.

The present findings extend previous work on IL-13 R130Q by linking the variant to airway smooth-muscle phenotypic modulation and remodeling-related outcomes across animal, cell, and computational models. However, the data remain preclinical and do not establish that IL-13 R130Q genotype should currently guide patient stratification or selection of anti-IL-13 therapy. Prospective human studies that incorporate genotype, airway remodeling measures, treatment response, and direct receptor-binding analyses will be required before clinical application can be considered.

This study has several limitations. First, total BALF cells were counted without differential leukocyte analysis; therefore, the contributions of eosinophils, neutrophils, lymphocytes, mast cells, basophils, and other populations remain undefined. Second, the *in vitro* experiments used commercially obtained primary hBSMCs from a single healthy donor. Cells from additional healthy donors and patients with asthma, including tissue obtained through biorepositories, are needed to establish donor-level reproducibility and disease-context relevance. Third, although previous studies support IL-4 and IL-5 release from human airway smooth muscle cells under IgE/FcϵRI activation or atopic-serum sensitization, IL-13-induced production of these cytokines has not been independently established. The present ELISA findings require confirmation using additional donors, IL4 and IL5 mRNA measurements, cell-free reagent controls, and independent protein-detection methods. Fourth, CCK-8 reflects cellular metabolic activity and was not complemented by EdU incorporation, Ki67 staining, CFSE dilution, or direct cell counting. Fluo-3/AM fluorescence reflects intracellular calcium signaling rather than direct contractile force, and the 24-h Boyden chamber assay does not demonstrate ASM migration *in vivo*. Fifth, recombinant human IL-13 was administered to mice, and possible species-specific differences should be considered. Sixth, JAK/STAT6 inhibitor experiments were not performed, and the IL-13Rα2 hypothesis was not functionally tested by receptor knockdown, overexpression, blocking, or direct binding assays. IL-13Rα1 binding was neither simulated nor measured, and receptor expression was not experimentally confirmed in the hBSMC preparation. Finally, the study did not assess combined IL-13 130R and IL-13 130Q exposure, and potential additive or synergistic effects remain unknown.

In conclusion, IL-13 130Q produced stronger remodeling-related and inflammatory effects than IL-13 130R in an OVA-induced type 2 allergic environment and induced greater CCK-8-derived proliferative/metabolic activity, intracellular calcium signaling, migration, cytokine concentrations, and JAK2/STAT6 phosphorylation in hBSMCs. Computational analyses further suggested reduced predicted stability of the IL-13 130Q/IL-13Rα2 complex. These findings support a context-dependent potentiating role for IL-13 130Q in airway smooth-muscle phenotypic modulation and airway remodeling. Direct receptor-binding studies, receptor-specific functional perturbation, inhibitor experiments, additional cell donors, and clinical validation are required to establish the underlying mechanism and translational relevance.

## Data Availability

The raw data supporting the conclusions of this article will be made available by the authors, without undue reservation.
